# Exposure of *Cryptococcus neoformans* to Seven Commonly Used Agricultural Azole Fungicides Induces Resistance to Fluconazole as Well as Cross-Resistance to Voriconazole, Posaconazole, Itraconazole and Isavuconazole

**DOI:** 10.3390/pathogens12050662

**Published:** 2023-04-29

**Authors:** Pascal Drakulovski, Donika Krasteva, Virginie Bellet, Sylvie Randazzo, Frédéric Roger, Cyrille Pottier, Sébastien Bertout

**Affiliations:** Laboratoire de Parasitologie et Mycologie Médicale, UMI 233 TransVIHMI, University of Montpellier, IRD, INSERM U1175, 15 Avenue Charles Flahaut, 34093 Montpellier, France

**Keywords:** *Cryptococcus neoformans*, pesticide, resistance, fluconazole, fungicide

## Abstract

Background: Cross-resistance to medical azoles by exposure to azole pesticides is well documented for *Aspergillus* family fungi but is poorly evaluated for other environmental pathogen fungi, particularly for yeasts belonging to the *Cryptococcus neoformans*/*Cryptococcus gattii* species complexes. Methods: One thousand *C. neoformans* yeast were exposed to various concentrations of seven different commonly used azole pesticides. Clones surviving exposure were picked randomly, and their minimal inhibitory concentrations (MICs) of fluconazole, voriconazole, posaconazole, itraconazole and isavuconazole were assessed. Results: Depending on the pesticide used for exposure, up to 13.3% of selected *Cryptococcus* colonies showed a phenotype of resistance to fluconazole, and among them, several showed cross-resistance to another or several other medical azoles. Molecular mechanisms involved in the resistance setups seem to be dependent on ERG11 and AFR1 gene overexpression. Conclusion: Exposure to any of the seven azole pesticides tested is capable of increasing the MIC of fluconazole in *C. neoformans*, including up to the level of the fluconazole-resistant phenotype, as well as generating cross-resistance to other medical azoles in some cases.

## 1. Introduction

The use of agricultural pesticides such as herbicides, insecticides, bactericides, molluscicides, nematicides or fungicides has greatly increased worldwide in recent years [1]. However, this overuse is not without side effects. Negative impacts on human health, with suspected or demonstrated links between exposure to pesticides and several acute and chronic diseases, are now widely recognized [2,3,4]. Pesticides are also suspected to be a major factor leading to biodiversity erosion [5,6,7]. Recently, another insidious deleterious effect has been described. Overuse or uncontrolled use of fungicides has been shown to be responsible for the appearance of cross-resistance to clinical antifungals among environmental fungi from the *Aspergillus* family. *Aspergillus* spp. are filamentous fungi that can lead to severe infections in immunocompromised patients. One of the main resistance mechanisms to azole family antifungals in *Aspergillus* is mediated by point mutations in the CYP51A gene, which encodes 14-α-demethylase, a key enzyme of the fungal ergosterol biosynthesis pathway [8,9,10]. Exposure of fungi from the *Aspergillus* family to agricultural azole pesticides during field treatments is now widely documented as a possible source for acquisition of these mutations in the CYP51 gene [11,12,13,14]. This may lead to cross-resistance to voriconazole, the first line of treatment against invasive aspergillosis, including in patients naïve for this molecule [15,16,17,18]. However, thus far, this phenomenon has not been demonstrated for the other environmental fungi that can become pathogenic in exposed populations. Indeed, *Coccidioides* spp., *Sporothrix* spp., *Cryptococcus* spp. and *Mucor* and *Rhizopus* spp. are all fungal pathogens present in the environment, soil and plants and have already been described as responsible for human infections. Among the aforementioned fungi, the most widespread in human pathology are the yeast from the *Cryptococcus neoformans* and *Cryptococcus gattii* species complexes. *C. neoformans* and *C. gattii* are environmental yeasts found in bird feces [19,20], some insects [21,22], decaying wood [20,23] and various plants, including cultured grapes [24]. As such, they are quite likely to be present in crops that are attractive for avian fauna. These fungi are responsible for cryptococcosis, an infectious disease that leads in most clinical cases to cryptococcal meningitis, a syndrome with a high death rate ranging from 10 to 70% [25]. This is especially true in resource-poor countries with a high HIV immunocompromised population. There are approximately 112,000 annual fatalities from this disease worldwide, with most occurring in Sub-Saharan Africa [26]. Care of cryptococcosis in this region of the world is complicated by limited resources and availability of treatments. Indeed, the main antifungal widely available to practitioners is fluconazole in the majority of care centers [27]. At the same time, the use of pesticides, including azole fungicides, is increasing in Sub-Saharan countries, especially on cash crops (cocoa, cotton, soybean, coffee, rubber, etc.), which are some of their main financial resources [28]. This relatively large use of pesticides comes with technical shortcomings. Use of obsolete molecules, inappropriate use or stockpiling, diversion of use, lack of financial and human resources, and lack of well-equipped laboratories for control and evaluation were documented, among other things [28,29,30]. Thus, the possibility of these yeasts acquiring resistance to medical azoles through exposure to agricultural azoles in the field cannot be excluded. Indirect clues about this possibility have emerged in various studies. Previous studies in Western Africa have shown that strains already resistant to fluconazole or exhibiting an elevated fluconazole MIC were present in patients naïve for this molecule in Cameroon [31,32,33], Ivory Coast [34] and DRC [35]. From a general point of view, there is increasing FCZ resistance in *Cryptococcus* strains isolated from Africa [36,37,38,39]. Causes can be multiple, from continuous host exposure to fluconazole during the course of infection [40] to poor compliance with the treatments either for antifungals or HAART or faulty host defenses and location of infection [41]. Preexposure of *Cryptococcus* strains to azoles in the environment could be a supplementary issue. With that aim in mind, the authors tested whether in vitro exposure of *Cryptococcus neoformans* to seven commonly used azole pesticides (difenoconazole, epoxyconazole, penconazole, tebuconazole, metconazole, bromuconazole and prothioconazole) could lead to resistance to medical triazole molecules, voriconazole, posaconazole, itraconazole, isavuconazole and mainly fluconazole, the main treatment widely available in most Sub-Saharan countries, and if so, through which mechanisms.

## 2. Materials and Methods

### 2.1. Strains

The strains used for this study were *Cryptococcus neoformans* serotype A H99 (ATCC 208821), *Candida parapsilosis* (ATCC 22019) and *Candida krusei* (ATCC 6258), which were used as quality control strains for antifungal susceptibility testing. Cultures were maintained on Sabouraud agar plates at 37 °C.

### 2.2. Azole Antifungals

Triazole antifungal powders, difenoconazole (DFZ), epoxyconazole (EPZ), penconazole (PNZ), tebuconazole (TBZ), metconazole (MTZ), bromuconazole (BMZ), prothioconazole (PTZ), fluconazole (FCZ), voriconazole (VOR), itraconazole (ITR), posaconazole (POZ) and isavuconazole (ISZ) were obtained from Sigma Aldrich, France.

### 2.3. In Vitro Exposure to Antifungal Pesticides

To mimic field exposure to the azole pesticides, 10^3^ *Cryptococcus neoformans* H99 cells in suspension in 100 µL of sodium saline solution were plated on Sabouraud agar plates and left to adsorb for one hour. For each given antifungal, solutions were prepared in DMSO with quantities corresponding to the highest commercial concentrations used during crop spraying as indicated by pesticide-providing companies (Table 1) scaled down to correspond to the surface of the agar plates. These quantities represented 100% of the deposit used in the field. A decreasing range of 100%, 75%, 50%, 25%, 15%, 10%, 5% and 2.5% of such concentrations was set up for each pesticide, and antifungal solutions were poured on the plates. Cultures were then grown at 25 °C for 5 to 7 days under a natural light cycle. A plate of yeasts treated under the same conditions with DMSO solution devoid of any pesticide was used as a control. The experiment was repeated three times for each pesticide molecule.

### 2.4. Antifungal Susceptibility Testing

Ten random colonies for each of the three repeated experiments were retrieved on each plate when significant growth (at least approximately 5% growth, i.e., 50 colonies when compared with the 10^3^ colonies in the untreated control) was observable for each antifungal molecule. Their in vitro susceptibility profiles against the 7 azole agropesticides listed above (including the pesticide they were generated with) as well as against fluconazole (FCZ), voriconazole (VOR), posaconazole (POS), itraconazole (ITR) and isavuconazole (ISA) were determined using the reference broth microdilution method in accordance with document M27-A4 of the Clinical and Laboratory Standards Institute (CLSI). Minimal inhibitory concentrations (MICs) for all molecules were defined as concentrations causing a 50% reduction in turbidity compared to the growth of the control at 72 h. *Candida krusei* ATCC 6258 and *Candida parapsilosis* ATCC 22019 were used as control strains as defined in the CLSI M27 Ed4 [42]. MIC assessment for each clone and each antifungal molecule was performed in duplicate (i.e., 2 × 2 readings). The mean MIC value by antifungal was obtained by averaging the four MIC values obtained for each antifungal molecule and each clone.

For the *C. neoformans* species complex, no break-point is available. Epidemiological cut-off (ECV) values to discriminate wild-type strains from strains with reduced susceptibility to antifungals (i.e., resistant phenotype) were used for the medical azole molecules with the following values: ECV (FCZ) = 8 µg/mL, ECV (POS) = 0.25 µg/mL, ECV (ISA) = 0.06 µg/mL, ECV (ITR) = 0.25 µg/mL and ECV (VOR) = 0.12 µg/mL [43,44,45,46]. Unfortunately, as such values do not exist for pesticides, no such classification could be conducted in regard to agricultural azoles.

### 2.5. ERG11 Gene Mutation Assessment

DNA was extracted for all strains showing a phenotype of resistance to fluconazole as follows: Cultures were retrieved directly from plates where they were grown in the presence of their respective agrochemicals used for selection and were incubated for 30 min at 75 °C in 200 µL of Cell Lysis Buffer Solution (Promega, Charbonnière-les-Bains, France) to which 25 µL of Proteinase K solution (Promega) was added. After incubation, 200 µL of BQ1 buffer was added (QuickPure NucleoSpin Blood Kit, Macherey Nagel, Hoerdt, France) and the whole extract was lysed with 0.22–0.25 mm silica beads by shaking in a Magnalyser apparatus (6 cycles of 40 s, 6000 rpm with cooling on ice between each cycle). Two hundred microliters of 100% ethanol was added, and the supernatant was retrieved and processed using silica columns (QuickPure NucleoSpin Blood Kit, Macherey Nagel) following the manufacturer’s instructions. The purity and quantity of DNA were assessed by spectrophotometry, and the DNA templates were used for PCR amplification of the ERG11 gene for all strains with Platinum PCR Supermix (Invitrogen/ThermoFisher Scientific, Villebon-sur-Yvette, France) and the primer pairs ERG11 Forward 5’ATG TCG GCA ATC ATC CCC CA3’ and ERG11 Reverse 5’GTG TTC GTG CTA CTC AAA TC3’. The conditions used were as follows: denaturation step of 5 min at 94 °C, 30 amplification steps (92 °C, 1 min; 60 °C, 1 min; and 72 °C, 2 min) and a final step of elongation of 72 °C for 5 min. The PCR products were then separated by migration on 1% agarose TAE gels, stained with ethidium bromide and visualized under UV. All samples were sent for sequencing to Genewiz, UK.

### 2.6. mRNA Expression Level Quantification

One hundred milligrams of fresh culture of *Cryptococcus* cells cultured on solid medium with or without their agrochemical of selection as described above was used for total RNA extraction with the Ribopure-Yeast Kit under the manufacturer’s specifications (Ambion/ThermoFisher Scientific, Villebon-sur-Yvette, France). The quantity of retrieved tRNA was measured and adjusted to perform further cDNA synthesis with the same amount of tRNA (1 µg) for each sample. cDNA was synthesized using the Superscript VILO cDNA synthesis kit (Invitrogen) as recommended by the manufacturer. qPCR was performed in 96-well plates using the LightCycler 480 SYBR kit within a LightCycler LC480 apparatus (Roche, Meylan, France) ) for the four genes of interest described as involved in FCZ resistance in *Cryptococcus* (ERG11, AFR1, AFR2, MDR1) as well as the household encoding gene for actin (ACT) and for all conditions (with or without agrochemical). The primers used were as follows:

ERG11 Forward: 5’ CCATGTCCGAGCTCATCATTCTT3’, ERG11 Reverse: 5’ ACTGGGAAGGGGCAAGTTGG3’; AFR1 Forward: 5’ TTCCCTGCTCCTTCAGGACAGA 3’; AFR1 Reverse: 5’ AGGCTTGGCCAGTTCGGTACT 3’; AFR2 Forward: 5’ CGATATGGGATTTCACTGCCCT 3’; AFR2 Reverse: 5’ GCTCCTGATATTTGTCGCTCTGC 3’; MDR1 Forward: 5’ GTCTTCACCTTCGTCCCGGAT3’; MDR1 Reverse: 5’ CAGTACTCTCACTCCCGGCCT G3’; ACT Forward: 5’ CCAAGCAGAACCGAGAGAAGATG 3’; ACT Reverse: 5’ GGACAGTGTGGGTGACAC CGT 3’.

The PCR parameters were as follows: heating/denaturation 600 s at 95 °C, 40 cycles of amplification (10 s, 95 °C; 15 s, 57 °C; and 15 s, 72 °C, single acquisition mode) and melting curve (95 °C, 10 s; 65 °C, 30 s; and 97 °C, 15 s, continuous acquisition mode). The level of expression was measured with automatic correction with LightCycler Software 4.05 (Roche) by relative quantification in comparison with the household gene and the ∆∆Cq method. All experiments were performed at least 2 times separately for each gene and each strain.

## 3. Results

### 3.1. Mean Growth in Regard to the Pesticide Concentration Range

Each pesticide had a different effect on the growth of the H99 cell inoculum when compared to untreated controls and resulted in significant growth (≥5% of the initial inoculum of 1000 cells) at different concentrations (Table 2).

For PNZ, 14.4% of H99 cells were able to grow when exposed to 75% of the advised commercial concentration, the equivalent of 75 g/L/Ha.

For BMZ, 4.9% growth was observed for 25% of the advised commercial concentration, the equivalent of 50 g/L/Ha.

For TBZ, 6.15% of growth was present with 15% of the advised concentration, so the equivalent of 37.5 g/L/Ha.

For 10% of EPZ, MTZ and PTZ, 5.15, 5.6 and 5.7% growth was observed, respectively. The equivalent quantities of advised commercial concentrations were 12.5 g/L/Ha, 9 g/L/ha and 20 g/L/Ha, respectively.

Finally, 9.3% growth was observed for 5% DFZ, the equivalent of 6.25 g/L/Ha.

### 3.2. Increase in MIC of FCZ after Exposure to Pesticide

The effect on the MIC of FCZ was also different for each pesticide (Table 3).

Analysis of the distribution of MIC (FCZ) for 30 colonies exposed to agricultural pesticide when compared with the MIC (FCZ) range of the H99 reference strain (0.5–2 µg/mL) indicates that some colonies had an increased MIC (4 to 64 µg/mL).

For DFZ, 86.6% (26/30) of the colonies had an MIC in the range of the reference strain, and 13.3% (4/30) had an elevated MIC in comparison with the reference strain. Among these, three colonies (10% of the overall tested population) had an MIC ≥ 16 µg/mL and a resistant phenotype in regard to FCZ.

For EPZ, 73.3% (22/30) had an MIC in the same range as the reference strain, and 26.6% (8/30) had an elevated MIC for FCZ. Among these, two colonies (6.6% of all tested colonies) had a phenotype of resistance to FCZ.

For PNZ, 73.3% (22/30) had an MIC similar to that of the reference strain, and 26.6% (8/30) showed an elevated MIC of FCZ. Three colonies (10% of the whole population) showed a phenotype of resistance to FCZ.

For TBZ, 83.3% (25/30) had an MIC of FCZ similar to that of the reference strain, and 16.6% (5/30) showed an elevated MIC, with only one having a phenotype of resistance to FCZ.

For BMZ, 73.3% (22/30) had an MIC similar to that of the H99 strain, and 26.6% (8/30) had an elevated MIC in comparison to that of the reference strain. Ten percent (3/30) of the colonies showed an MIC above the epidemiological cut-off, indicating a phenotype of resistance to FCZ.

For MTZ, 86.6% (26/30) showed an MIC in the range of the H99 strain, and 13.3% (4/30) of colonies showed an elevated MIC of FCZ, with a single colony bearing a phenotype of resistance to that antifungal.

For PTZ, 76.6% (23/30) had an MIC comparable to that of the reference strain, and 23.3% (7/30) of the colonies had an increased MIC in comparison to that of H99, with four colonies (13.3% of the whole pool tested) showing a phenotype of resistance to FCZ.

Comparison of the mean FCZ MIC values for each group of 30 colonies selected from cultures exposed to agropesticides gave the following ranking: PNZ > PTZ > EPZ > BMZ > TBZ > MTZ > DFZ.

This indicated that the highest FCZ MIC level was achieved when the yeasts were exposed to PNZ, and the lowest FCZ MIC level was obtained when the yeasts were exposed to DFZ.

### 3.3. Increase in MIC of Other Azoles

All colonies with a phenotype of resistance to FCZ, three obtained by contact with DFZ (clones DFZ5, DFZ6 and DFZ7), two with EPZ (clones EPZ20 and EPZ29), three with PNZ (clones PNZ3, PNZ29 and PNZ30), one with TBZ (clone TBZ14), one with MTZ (clone MTZ11), three with BMZ (clones BMZ5, BMZ19, BMZ30) and four with PTZ (clones PTZ4, PTZ7, PTZ8, PTZ9), were tested by CLSI for their MICs of four medical azoles (Table 4) as well as the seven agricultural pesticides (Supplementary data S1).

One phenotype of resistance to FCZ on three (33.3%) obtained from DFZ plates also showed a resistant phenotype for ISV (clone DFZ7).

One phenotype of resistance to FCZ (50%) on two obtained from EPZ plates showed a resistant phenotype for ISV (clone EPZ20).

One phenotype of resistance to FCZ on three (33.3%) obtained from PNZ plates showed a resistant phenotype for both ITR and ISV (clone PNZ3).

The only colony with a phenotype of resistance to FCZ obtained with TBZ also showed a phenotype of resistance to all four other medical azoles (clone TBZ14).

The only clone resistant to FCZ obtained with MTZ showed a phenotype of resistance to POZ (clone MTZ11).

No colonies resistant to FCZ selected by BMZ exposure showed another resistant phenotype against any other medical azole.

Finally, one clone with a phenotype of resistance to FCZ obtained from PTZ plates (1/4, 25%, clone PTZ4) also showed a phenotype of resistance to ISV, and another clone resistant to FCZ, different from the previous one (1/4, 25%), showed a resistant phenotype against POZ and VOR (clone PTZ9).

### 3.4. Theorical Field Surface where Colonies with Elevated FCZ MIC May Appear

**Figure 1:**
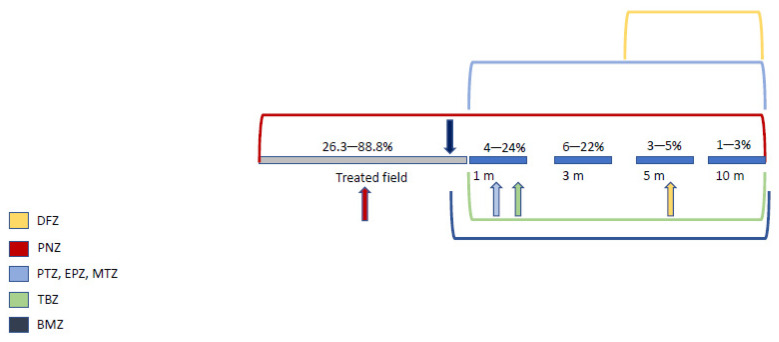
Hypothetical distance from spraying point of pesticides where the colonies of *C. neoformans* with a high MIC of FCZ may appear. Distance expressed in meters indicates the distance from the spraying point as per Druart et al., 2011 [47]. Percentage indicates the amount of deposit of tebuconazole according to the distance as per Druart et al., 2011 [47]. Brackets indicate the area where pesticide deposit may be enough to allow both a significant survival of potentially present yeasts (≥5% survival after treatment) and the appearance of colonies with high FCZ MIC for each pesticide tested. Arrows indicate the first distance to the spraying point where colonies with high FCZ MIC may appear for each pesticide tested. DFZ = difenoconazole, EPZ = epoxyconazole, PNZ = penconazole, TBZ = tebuconazole, MTZ = metconazole, BMZ = bromuconazole, PTZ = prothioconazole.

According to various studies on TBZ, spray drift and pesticide dispersal can generate a decrease of 11 to 74% of the pesticide concentration in treated fields [47,48]. Furthermore, spray drift could send a small amount of pesticide beyond the treatment area [47]. Based on the previous results of TBZ dispersal and the concentrations generating colonies with high FCZ MIC for each of the seven molecules tested (Table 2), we propose a model of the areas where such colonies with high FCZ MIC may appear in the field by molecule (Figure 1).

For PNZ, the first significant growth (≥5%) was observed at 75% of the deposit of the commercially advised concentration, indicating that the whole surface of any treated field may be at risk of seeing colonies with high FCZ MIC appear.

For PTZ, EPZ and MTZ, the first significant growth (≥5%) was observed at 10% of the deposit, meaning that the risk of emergence of resistant colonies at higher concentrations is limited. For these three molecules, in-field concentrations would theoretically not allow the growth of colonies with high MIC of FCZ directly in the treated area. However, according to Druart et al. [47], 10% of the deposits of the treatment could be observed at 0 to 3 m beyond the spraying point for TBZ. This suggests that it is the peripheral surface of any field treated with one of these three pesticides that may be most at risk of seeing potentially present *Cryptococcus* yeasts develop an elevated MIC of FCZ.

For TBZ, the first significant growth was observed at 15% of the deposit, which gives roughly the same pattern as the three other pesticides mentioned above.

For DFZ, the first significant growth was observed at 5% of the deposit, which corresponds, according to Druart et al., to the concentrations obtained by spray drift in the 5 m fringe and beyond of a treated field.

Finally, for BMZ, the first significant growth was observed at 25% of the deposit. This concentration could be found in the immediate peripheral area of the field due to spray loss and spray drift (Figure 1).

### 3.5. Assessment of ERG11 Point Mutations

No ERG11 mutations were found in any of the strains with a phenotype of resistance to FCZ regardless of the agropesticides they were obtained with.

### 3.6. Gene Expression Quantification

**Figure 2:**
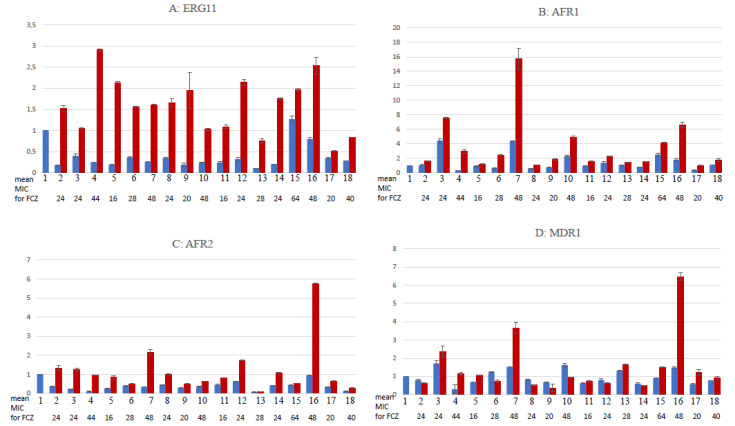
Expression levels of ERG11, AFR1, AFR2 and MDR1 of the various clones with an MIC of fluconazole >8 µg/mL. Levels of expression of ERG11 (**A**), AFR1 (**B**), AFR2 (**C**) and MDR1 (**D**) were evaluated in comparison with the expression of these same genes in the H99 control strain, in the absence (blue bars) or presence (red bars) of the pesticide used to obtain the tested isolates. Tested isolates are numbered as follows: 1: H99 control strain, 2: DFZ 5; 3: DFZ 6; 4: DFZ 7; 5: EPZ 20; 6: EPZ 29; 7: PNZ 3; 8: PNZ 29; 9: PNZ 30; 10: TBZ 14; 11: MTZ 11; 12: BMZ 5; 13: BMZ 19; 14: BMZ 30; 15: PTZ 4; 16: PTZ 7; 17: PTZ 8; 18: PTZ 9. FCZ MIC line indicates the mean fluconazole MIC of the various clones. DFZ = difenoconazole, EPZ = epoxyconazole, PNZ = penconazole, TBZ = tebuconazole, MTZ = metconazole, BMZ = bromuconazole, PTZ = prothioconazole, FCZ = fluconazole.

Gene expression for ERG11, AFR1, AFR2 and MDR genes was assessed for all clones with an MIC (FCZ) > 16 µg/mL in the presence or absence of the agropesticide that allowed their selection. Gene expression levels were obtained by comparison with the H99 control strain and are available in Figure 2 and Supplementary Data S2. Regardless of the agropesticide initially used to obtain the strain, ERG11 expression was lower for 17/17 strains than for the H99 control strain when growth was performed without agrochemical antifungals. ERG11 expression levels increased for all strains (up to 11.6×”) when the strains were grown with their initial pesticide of selection. The same trend was observed for AFR1 and AFR2, with an expression level lower than or equal to that present in the control strain in the absence of pesticide and an increase in expression for all clones (up to 8.45× and 9.4× for AFR1 and AFR2, respectively) in the presence of the pesticide of selection. The results were more irregular with MDR1 expression levels, with 11/17 clones showing underexpression and 6/17 showing overexpression of MDR when compared with the H99 strain in the absence of pesticide. When grown in the presence of their selection pesticides, 10 strains (10/17) showed increased expression (up to 3.9×), while 8 strains (7/17) showed decreased expression (down to 1.7×) of MDR.

## 4. Discussion

Very few studies have assessed whether pesticide-mediated resistance to medical azole antifungals can exist for pathogenic environmental fungi other than *Aspergillus* spp. To date, a single article has referred to the fact that exposure of *C. neoformans* and *C. gattii* to tebuconazole can lead to cross-resistance to fluconazole, itraconazole and ravuconazole [49]. However, this possible resistance to clinical molecules in pathogenic yeasts regularly isolated from plants and bird feces is of high interest, especially in countries with a high cryptococcosis burden due to a large HIV population pool and poor access to molecules other than fluconazole for the care of cryptococcosis infections. The most exposed region of the world is the Sub-Saharan region, with the majority of lethal cases of CNM registered [26]. This is also a region where the use of agropesticides was historically more limited than in northern countries. However, the use of pesticides is increasing due to the importance of cash crops for the economies of the various countries in this area [28,50]. The combination of all these factors, including a large immunodeficient population pool, poor access to molecules other than fluconazole, favorable environmental conditions for *Cryptococcus* survival and increased use of agropesticides, makes these countries particularly vulnerable if such a phenomenon is proven possible.

### 4.1. Gradation of Risk of Generating Strains with High MIC of FCZ by Pesticide

Among the seven molecules tested, BMZ, EPZ and PNZ generated the highest number of elevated MIC (FCZ) colonies among those picked for testing. Furthermore, PNZ and BMZ gave 10% of colonies with a phenotype of resistance to FCZ (MIC > 8 µg/mL), while in the case of EPZ, this concerned fewer colonies. PTZ preexposure generated fewer elevated MIC (FCZ) colonies but more colonies with a resistant phenotype than the three aforementioned molecules. The molecules to which preexposure generated the lowest number of elevated MIC (FCZ) colonies were TBZ, MTZ and DFZ. Moreover, TBZ and MTZ generated very few clones with a true phenotype of resistance to FCZ. DFZ, on the contrary, generated colonies with a phenotype of resistance to FCZ in a number similar to what was observable with PNZ and BMZ. In order of importance for the number of FCZ-resistant phenotype clones generated, which is the first concern for care in patients, as per our results, the molecules can be ranked as follows: PTZ > BMZ = PNZ = DFZ > EPZ > TBZ = MTZ. However, increased MIC colonies, even if below the resistance value, should not be discarded, as an elevated MIC of FCZ could be a predictive factor for a negative outcome during patient care [51]. Indeed, this may be a sign that a resistance mechanism is currently in the process of taking place, possibly leading to future therapeutic failure. This is particularly true in countries from the Sub-Saharan region where first-line treatment is mostly performed with FCZ and may sometimes be administered at suboptimal doses of 200–400 mg/day [52,53,54]. In these situations of low-dose monotherapy, resistance acquisition is already problematic [54] and could be worsened by preadaptation of the yeasts to the treatment during field exposure to azole pesticides. This situation could also remain true even with the more widely adopted 1200 mg of FCZ for 2 weeks of induction followed by an 800 mg/day regimen for consolidation monotherapies [55]. From that point of view, in regard to the overall number of elevated MIC clones generated by preexposure to agricultural azoles, the ranking of the seven pesticides tested in terms of further possible risk for therapeutic failure due to emerging resistance is BMZ = EPZ = PNZ > PTZ > TBZ > MTZ = DFZ.

Regardless of the molecule tested, it appears that preexposure to azole pesticides allows the emergence of various numbers of colonies with an FCZ-resistant phenotype in proportions from 3.3% to 10%, which can be compared with the percentage of patients showing a resistant strain at the beginning of their treatment with FCZ in the various African studies already mentioned. It is also important to note that in a previous study, Bastos et al. [49] showed that cross-resistance from TBZ to a medical azole needed an adaptation period. In our study, most results were obtained after short-term exposure (5–7 days). It cannot be excluded that a longer or iterative exposure increases the proportion of elevated FCZ MIC and FCZ-resistant phenotype yeasts. This is especially important to keep in mind knowing that manufacturers’ advice for use in horticulture or agriculture suggests two sprayings of azole pesticides at 14–21-day intervals most of the time.

### 4.2. Cross-Resistance to Other Medical Azoles

Among the colonies with a fluconazole-resistant phenotype obtained by exposure to azole pesticides, several also showed a phenotype of resistance to at least one other medical azole among the following: posaconazole, itraconazole, voriconazole and ravuconazole. Cross-resistance to other medical azoles in *Cryptococcus* strains with an already high FCZ MIC is not unheard of [56,57] but remains rare. In contrast, in this study, we observed a high amount of cross-resistance with 8/17 isolates (47%), including one isolate obtained by exposure to tebuconazole showing a phenotype of resistance to all medical azoles tested. Resistance to medical azoles in *Cryptococcus* can be mediated mainly by two mechanisms. The first mechanism is point mutations in the ERG11 gene leading to the amino acid substitution G484S [58] or G344S [57]. However, none of the isolates with high FCZ MIC selected by pesticide contact showed a point mutation in their ERG11 gene here. The second azole-resistance mechanism is the overexpression of AFR1, AFR2 or MDR1 efflux pump-encoding genes [59]. Such overexpression was found for 5 out of 17 isolates when such isolates were grown without any pesticide. When the pesticide of selection was added, all isolates (17/17) experienced overexpression of one or several of these genes. ERG11 and AFR1 experienced an increase in expression in all clones in the presence of pesticides, while AFR2 showed an increase in expression in 16/17 clones. Concerning MDR1, only 10 clones showed increased expression in the presence of pesticide. This suggests that one of the main mechanisms for FCZ MIC elevation in our study is linked mostly to the ERG11 and AFR1 genes and more likely a combination of both of them, as no high FCZ MIC clones had either gene overexpressed alone. The involvement of AFR2 and MDR1 may be secondary in our case. These results strongly suggest that any of the seven agrochemicals tested may act as inducers of overexpression of ERG11, AFR1, AFR2 or MDR. This was already shown for tebuconazole [49] but not for any of the six other pesticides. It also confirms the role of exposure to azole pesticides in triggering cellular mechanisms that may lead to an increase in the MIC of medical azoles in environmental fungi. However, no correlation was found between the level of overexpression of any gene and the MIC of FCZ. This suggests the possible involvement of other mechanisms in the MIC increase alongside the overexpression of ERG11 and AFR. The cell functions involved could be heteroploidy [60], cell wall enlargement [61] or alternate transporters not tested here [62,63].

### 4.3. Estimation of Field Areas at Risk of Generating High-MIC Fungi

As preexposure to azole pesticides seems to trigger MIC (FCZ) increases, including up to the resistance range, it is also important to try to evaluate the relative amount of population and soil surface exposed to this phenomenon. Unfortunately, data about the use of pesticides in Sub-Saharan Africa are scarce, with the exception of South Africa, which will be used as the reference situation in further discussion [64]. Azole pesticides are known to be stable molecules that are poorly impacted by photolysis and poorly mobile in the soil and thus have a long half-life [65,66,67]. However, depending on the nature of the molecule used, its solvent, the mean dispersal and the environmental conditions at the moment of spraying, various studies have shown that no pesticide reaches 100% deposition on plant material or soil. Losses and unwanted dispersal by spray drift can be high. Those losses can reach 11% to 74% of the initial doses, as shown for tebuconazole in one study [47]. The variation in deposition ranged from 53.9 to 80% for the same triazole in another study [48]. This can, of course, have a consequence on the quantity of pesticide coming into contact with potential environmental fungi, allowing it to reach the theoretical resistance-inducing concentrations observed in this study. Spray drift deposits can also allow pesticides to reach beyond the treated fields, expanding the areas at risk for generating resistance. By using a model based on the data established by Druart et al. [47], it was estimated that the area where colonies of *Cryptococcus* with high MICs of FCZ possibly emerged in fields was the whole sprayed area for PNZ; the immediate fringe of the sprayed area for BMZ; the peripherical area of the treated fields (from 1 m and beyond) for PTZ, EPZ, MTZ and TBZ; and the distant fringe (from 5 m and beyond) of sprayed surfaces for DFZ. Once these theoretical risk areas are defined, it is important to evaluate the number of agriculture workers exposed to each pesticide. PNZ, for example, is largely used in grapevine cultures in South Africa; this is a culture that still requires grape pickers, which are often a vulnerable population for health issues, including HIV status, because of their poverty and migratory and daily worker status [50]. PTZ is registered in South Africa for use on wheat and barley, which are cultures requiring fewer farm hands and are more mechanized [64] but cover large zones and thus also have large peripherical surfaces. These cultures are also known to attract birds that are the main reservoir for *C. neoformans* complex species, and *Cryptococcus* yeast has already been described in such culture phyllospheres [68]. It should also be noted that this molecule is registered for vegetables, but whether it is used for that purpose in Africa remains unclear. EPZ is a molecule registered for maize, corn, wheat, barley and groundnuts/peanuts in South Africa with the same issues as PTZ. MTZ is less widely used than other azole pesticides, mainly on grain crops and as a means to control mycotoxigenic *Fusarium* [69]. In contrast, TBZ is very widely used on a large number of different types of cultures from potatoes, soya beans, bananas, mangoes, peas and grapes [64], increasing the risk of field contact between environmental fungi potentially pathogenic to humans and suboptimal or spray drift deposit concentrations, allowing an increase in the MIC of FCZ. This is particularly true for the *C gattii* species complex, which has already been isolated from mango trees [70], as well as *C. neoformans*, which has already been isolated from soybean-derived products [71]. BMZ is one of the three molecules that generated the highest number of colonies with elevated MIC of FCZ among all the pesticides tested. The results were observed at 25% of the deposit. This concentration could be found both in the field due to spray loss and in peripheral areas due to spray drift [47]. This enlarges the area where potential contact between *Cryptococcus* yeasts and pesticide deposit concentrations leads to further FCZ resistance. This molecule is registered for wheat and barley in South Africa but also for cultures such as apples and coffee [64]. Apples and coffee are labor-intensive cultures, using a large pool of workers and thus increasing the probability of human contact with a high-MIC *Cryptococcus* strain. Finally, DFZ showed significant growth at very low amounts of deposits (5%). This low deposit means that very few or no colonies are supposed to survive in a sprayed field even with loss of deposits on the soil and that the risk of emergence of resistant cryptococci exists only beyond 5 m from the spraying point. Unfortunately, this is also a distance commonly used to house land workers either temporarily during the harvesting season or permanently, which increases the probability of promoting contact between humans and resistance-acquiring *Cryptococcus* strains. This is especially of concern when knowing that this molecule is widely used in labor-intensive fruit crops such as pear, tomato or groundnut cultures requiring a large and readily available workforce. It should be noted that these estimations do not take into account the time and quality of storage, freshness of the molecules’ batches, management of pesticide stockpiles or methodology of application, which are known to be problematic in Sub-Saharan Africa [29]. Such mismanagements could reduce the concentration of antifungal products before application and thus increase the deposit or spray drift area where antifungal quantities would be insufficient to eradicate potential *Cryptococcus* sites but sufficient to trigger a setup of resistance mechanisms. Moreover, most African countries other than South Africa rely on less mechanization and more hand picking for their agricultural activities. Recent studies also suggest that pesticides are more frequently applied by African households than acknowledged and are not limited to high-value crops but are also on stable grain at the community/familial farmhouse levels, increasing the range of land and population exposed [72]. This also raises concern regarding the use of azole pesticides on stable grain within the range of nearby hen breeding, a regular case in African familial communities, because *Cryptococcus* yeasts have already been sourced from domestic poultry or pigeon cages [73,74]. Finally, this situation can also be of concern in countries basing a part of their economy on horticulture, such as Kenya, Ethiopia, Ghana and Uganda, because concentrations of pesticides and, in particular, antifungals in horticulture are usually lower than those in agriculture, meaning that the simulated deposit quantities leading to elevated MICs for FCZ in *Cryptococcus* yeasts assessed in this study could be reached more easily.

In conclusion, this in vitro study showed that short-term preexposure to any of the seven agricultural azoles tested generated high-MIC (FCZ) *Cryptococcus* strains with a proportion ranging from 3.3% for MTZ to 16.6% for BMZ. Deposit quantities of chemicals leading to these high-MIC *Cryptococcus* varied, meaning that the extrapolated risk area in the fields is not the same for each molecule. To precisely clarify this risk, real field tests and a search for high-MIC (FCZ) environmental strains should be performed.

## Figures and Tables

**Table 1:** Concentrations of use for the pesticides tested in this study advised by manufacturers.

Antifungal	Acronym	Highest Commercial Concentrations as Advertised by Manufacturers for Use on Fields in Agriculture
Tebuconazole	TBZ	250 g L Ha
Bromuconazole	BMZ	200 g L Ha
Prothioconazole	PTZ	200 g L Ha
Difenoconazole	DFZ	125 g L Ha
Epoxiconazole	EPZ	125 g L Ha
Penconazole	PNZ	100 g L Ha
Metconazole	MTZ	90 g L Ha

**Table 2:** Mean growth of an inoculum of 1000 H99 cells after 5–7-day exposure to various quantities of antifungal pesticides.

	Mean Growth of H99 Culture Exposed to Various Amounts of Antifungals in Comparison with an Untreated Control							
**Antifungal Molecule**	**100%**	**75%**	**50%**	**25%**	**15%**	**10%**	5%	2.5%
DFZ	0	0	0	0	0	0.1% (±0.05)	**9.3%** **(±2.7)**	64.8% (±11.3)
EPZ	0	0	0	0.06% (±0.03)	0.24% (±0.11)	**5.15%** **(±2.5)**	52.4% (±9.1)	
PNZ	2.5% (±1.3)	**14.4%** **(±7.2)**	57.2% (±15.9)	91.9% (±12.4)				
PTZ	0	0	0	0.06% (±0.02)	2.5% (±1.5)	**5.6%** **(±1.7)**	43.6% (±18.1)	
TBZ	0	0	0	2% (±0.23)	**6.7%** **(±3.1)**	35.8% (±11.9)	87.1% (±5.2)	
MTZ	0	0	0	0.4% (±0.09)	1.65% (±1.51)	**5.7%** **(±0.9)**	86% (±10.8)	
BMZ	0	0	0	**4.9%** **(±2.2)**	11.6% (±4.4)	57.2% (±13.9)	80.1% (±14.1)	

Concentration of 100% is the highest commercial concentration available for each specific antifungal for crop spraying as indicated by selling companies (Table 1) scaled down in proportion for correspondence with the surface of an agar plate. Numbers in bold indicate the first concentration with a significant growth (≥5%) from which 30 colonies were picked to be tested later against fluconazole. Number in brackets indicates the standard deviation in colony count in percentage.

**Table 3:** Distribution of 30 colonies after pesticide exposure according to their MIC of fluconazole. White columns show number of pesticide-exposed colonies with an FCZ MIC in the same range as the control strain.

	Distribution of 30 Colonies Selected by Exposure to Pesticides According to Their MIC of Fluconazole (µg/mL)			% of Colonies with Elevated MIC of FCZ	% of Colonies with a Phenotype of Resistance to FCZ
**Antifungal Molecules**	**0.5–2**	4–8	16–64		
DFZ	26	1	3	13.3%	10%
EPZ	22	6	2	26.6%	6.6%
PNZ	22	5	3	26.6%	10%
TBZ	25	4	1	16.6%	3.3%
MTZ	26	3	1	13.3%	3.3%
BMZ	22	5	3	26.6%	10%
PTZ	23	3	4	23.3%	13.3%

Deep gray shows number of pesticide-exposed colonies with an FCZ MIC higher than the H99 control strain FCZ MIC range (elevated MIC of FCZ). Red shows number of pesticides exposed colonies with FCZ MIC reaching the phenotype of resistance to FCZ. DFZ = difenoconazole, EPZ = epoxyconazole, PNZ = penconazole, TBZ = tebuconazole, MTZ = metconazole, BMZ = bromuconazole, PTZ = prothioconazole, FCZ = fluconazole.

**Table 4:** Mean MIC of medical azole molecules for all clones with a phenotype of resistance to fluconazole selected by agropesticide contact.

	Mean MIC ATF 72h				
Clones	FCZ	POZ	VOR	ITR	ISV
H99	0.5–2	0.125	0.06	0.125	<0.03
DFZ 5	24	<0.03	<0.03	<0.03	0.06
DFZ 6	24	<0.03	0.06	<0.03	<0.03
DFZ 7	44	0.25	0.0925	0.125	0.25
EPZ 20	16	<0.03	0.125	0.125	0.125
EPZ 29	28	0.06	<0.03	<0.03	<0.03
PNZ 3	48	<0.03	0.06	0.5	0.125
PNZ 29	24	0.06	<0.03	0.06	<0.03
PNZ 30	20	0.5	0.0925	0.1875	0.06
TBZ 14	48	1	0.25	1	0.5
MTZ 11	16	0.5	0.125	0.25	<0.03
BMZ 5	24	0.06	0.125	0.25	<0.03
BMZ 19	28	<0.03	<0.03	0.06	<0.03
BMZ 30	24	<0.03	0.06	0.06	<0.03
PTZ 4	64	0.25	0.125	0.25	0.125
PTZ 7	48	0.1875	0.06	0.125	<0.03
PTZ 8	20	<0.03	<0.03	<0.03	<0.03
PTZ 9	40	0.5	0.1875	0.25	0.045

MIC for each clone was assessed by the CLSI M27A4 broth microdilution method in duplicate. Mean MIC value is the mean score obtained by averaging the four separate MICs of any antifungal for each clone. Cells in red indicate the antifungals for which MIC is above the ECV for *Cryptococcus neoformans*. DFZ = difenoconazole, EPZ = epoxyconazole, PNZ = penconazole, TBZ = tebuconazole, MTZ = metconazole, BMZ = bromuconazole, PTZ = prothioconazole, FCZ = fluconazole, POZ = posaconazole, VOR = voriconazole, ITR = itraconazole and ISV = isavuconazole.

## Data Availability

Data are contained within the article or supplementary material. No sequences or strains were submitted to any database or library.

## Supplementary Materials

The following supporting information can be downloaded at: https://www.mdpi.com/article/10.3390/pathogens12050662/s1, Table S1: MICs of pesticide azole molecules for the clones with a resistant phenotype to FCZ selected by agropesticide contact; Table S2: Gene expression of ERG11, AFR1, AFR2 and MDR1 of the various clones with a MIC of fluconazole >8 µg/mL.
