# Supplementary material for: Exposure of Cryptococcus neoformans to Seven Commonly Used Agricultural Azole Fungicides Induces Resistance to Fluconazole as Well as Cross-Resistance to Voriconazole, Posaconazole, Itraconazole and Isavuconazole
Source: Pathogens. 2023 Apr 29;12(5):662. doi: 10.3390/pathogens12050662 (PMC10224184; doi:10.3390/pathogens12050662)
Supplement: Supplementary file 1 [file pathogens-12-00662-s001.zip › Supplementary Data S1BIS.pdf]

	Mean MIC ATF 72h						
Clones	DFZ	EPZ	PNZ	TBZ	MTZ	BMZ	PTZ
H99	<0.03	0.075	0.375	0.125	0.045	0.075	0.075
DFZ 5	<0.03	<0.03	<0.03	0.5	0.06	<0.03	0.5
DFZ 6	0.125	0.0925	1.5	1	0.125	0.75	0.5
DFZ 7	0.25	0.125	2	1	0.125	2	0.5
EPZ 20	0.125	0.125	1	0.375	0.125	0.5	0.187
EPZ 29	<0.03	0.125	0.06	0.125	0.06	<0.03	<0.03
PNZ 3	0.0925	<0.03	0.5	0.25	0.045	0.5	0.25
PNZ 29	0.045	<0.03	0.06	0.125	0.06	0.06	<0.03
PNZ 30	0.06	0.125	0.375	0.25	0.125	0.5	0.25
TBZ 14	0.125	0.125	3	1	0.25	2	0.5
MTZ 11	0.0925	0.06	1	1	0.125	1	1
BMZ 5	0.125	0.125	1	1	0.125	0.25	0.5-1
BMZ 19	<0.03	<0.03	0.125	<0.03	0.06	0.5	0.125
BMZ 30	<0.03	<0.03	0.5	0.125	<0.03	0.5	0.5
PTZ 4	0.125	0.125	1	0.5	0.06	1	0.125
PTZ 7	<0.03	<0.03	<0.03	<0.03	<0.03	0.125	<0.03
PTZ 8	0.06	0.045	0.0925	0.06	<0.03	0.06	<0.03
PTZ 9	0.1875	0.125	2	1	0.125	0.5	1

Table S1 : Supplementary Data

MICs of pesticide azole molecules for the clones with a resistant phenotype to FCZ selected by agropesticide contact

MIC for each clone was assessed by the CLSI M27A4 broth microdilution method in duplicate. Mean MIC value is the mean score obtained by averaging the four separate MIC to any antifungal for each clone.

Square in grey indicate indicate MIC values when the tested clone exposed to its antifungal of selection

DFZ =Difenoconazole, EPZ = Epoxyconazole, PNZ =Penconazole, TBZ = Tebuconazole, MTZ = Metconazole, BMZ = bromuconazole, PTZ= Prothioconazole
