# Supplementary material for: Exposure of Cryptococcus neoformans to Seven Commonly Used Agricultural Azole Fungicides Induces Resistance to Fluconazole as Well as Cross-Resistance to Voriconazole, Posaconazole, Itraconazole and Isavuconazole
Source: Pathogens. 2023 Apr 29;12(5):662. doi: 10.3390/pathogens12050662 (PMC10224184; doi:10.3390/pathogens12050662)
Supplement: Supplementary file 1 [file pathogens-12-00662-s001.zip › Supplementary Data S2BIS.pdf]

Clone	Mean MIC to FCZ of the clone	ERG11	AFR1	AFR2	MDR1
DFZ 5 - ATF	24	0,18+/-0,01	1,07+/-0,09	0,37+/-0,02	0,79+/-0,05
DFZ 5 + ATF	24	1,52+/-0,07	1,62+/-0,02	1,34+/-0,12	0,62+/-0,02
DFZ 6 – ATF	24	0,40+/-0,04	4,45+/-0,29	0,21+/-0,01	1,71+/-0,16
DFZ 6 + ATF	24	1,05+/-0,01	7,54+/-0,07	1,24+/-0,1	2,37+/-0,3
DFZ 7 -ATF	44	0,25+/-0,01	0,35+/-0,01	0,10+/-0,02	0,29+/-0,35
DFZ 7 + ATF	44	2,91+/-0,02	2,96+/-0,25	0,94+/-0,03	1,14+/-0,08
EPZ 20 – ATF	16	0,19+/-0,01	0,92+/-0,08	0,25+/-0,02	0,68+/-0,01
EPZ 20 + ATF	16	2,13+/-0,03	1,21+/-0,02	0,85+/-0,09	1,06+/-0,02
EPZ 29 -ATF	28	0,37+/-0,02	0,66+/-0,02	0,40+/-0,01	1,23+/-0,02
EPZ 29 + ATF	28	1,56+/-0,02	2,44+/-0,04	0,50+/-0,01	0,72+/-0,08
PNZ 3 - ATF	48	0,26+/-0,01	4,36+/-0,05	0,34+/-0	1,52+/-0,01
PNZ 3 + ATF	48	1,61+/-0,01	15,73+/-1,44	2,15+/-0,15	3,64+/-0,3
PNZ 29 – ATF	24	0,35+/-0,02	0,58+/-0,04	0,45+/-0,02	0,82+/-0,03
PNZ 29 + ATF	24	1,65+/-0,1	1,06+/-0,02	0,98+/-0,04	0,52+/-0,01
PNZ 30 - ATF	20	0,19+/-0,04	0,71+/-0,05	0,3+/-0,01	0,67+/-0,02
PNZ 30 + ATF	20	1,95+/-0,43	1,84+/-0,13	0,5+/-0,02	0,37+/-0,2
TBZ 14 - ATF	48	0,25+/-0,01	2,32+/-0,14	0,35+/-0,02	1,62+/-0,08
TBZ 14 + ATF	48	1,03+/-0,03	4,91+/-0,18	0,64+/-0	0,94+/-0,02
MTZ 11 – ATF	16	0,24+/-0,02	0,93+/-0,02	0,45+/-0,03	0,62+/-0,01
MTZ 11 + ATF	16	1,09+/-0,04	1,59+/-0,05	0,81+/-0,01	0,71+/-0,04
BMZ 5 - ATF	24	0,32+/-0,05	1,37+/-0,17	0,62+/-0,02	0,80+/-0,07
BMZ 5 + ATF	24	2,14+/-0,07	2,28+/-0,09	1,73+/-0,01	0,62+/-0,02
BMZ 19 – ATF	28	0,11+/-0,01	1,06+/-0,03	0,07+/-0,02	1,30+/-0,02
BMZ 19 + ATF	28	0,75+/-0,05	1,42+/-0,04	0,09+/-0,01	1,65+/-0,01
BMZ 30 – ATF	24	0,21+/-0,01	0,83+/-0,01	0,42+/-0,01	0,59+/-0,04
BMZ 30 + ATF	24	1,75+/-0,02	1,53+/-0,03	1,07+/-0,05	0,49+/-0,01
PTZ 4 - ATF	64	1,27+/-0,07	2,51+/-0,07	0,43+/-0,01	0,90+/-0,02
PTZ 4 + ATF	64	1,97+/-0,03	4,18+/-0,22	0,52+/-0,01	1,50+/-0,02
PTZ 7 - ATF	48	0,80+/-0,03	1,77+/-0,13	0,95+/-0,02	1,48+/-0,03
PTZ 7 + ATF	48	2,53+/-0,2	6,61+/-0,4	5,76+/-0,02	6,48+/-0,2

PTZ 8<br>- ATF	20	0,35+/-0,02	0,37+/-0,03	0,32+/-0,01	0,57+/-0,03
PTZ 8<br>+ ATF	20	0,52+/-0,01	1+/-0,04	0,65+/-0,02	1,22+/-0,14
PTZ 9<br>- ATF	40	0,29+/-0,01	1,06+/-0,01	0,12+/-0,02	0,77+/-0,01
PTZ 9<br>+ ATF	40	0,84+/-0,01	1,74+/-0,2	0,27+/-0,03	0,90+/-0,1

Table S2 Supplementary Data. Gene expression of ERG11, AFR1, AFR2 and MDR1 of the various clones with a MIC for fluconazole >8µg/mL.

Levels of expression of ERG11, AFR1, AFR2 and MDR1 were evaluated in comparison with the expression of these same genes in the H99 control strain, in absence (-ATF) or presence (+ATF) of the pesticide used to obtain the tested isolates.

Squares in red indicate an over expression of the gene when the triazole pesticide used for selecting the clone was added to the culture in comparison with culture grown without pesticide. Blue squares indicate a decreased gene expression when the triazole pesticide used for selecting the clone was added to the culture in comparison with culture grown without pesticide. Grey square indicates the same level of expression of the gene when the triazole pesticide used for selecting the clone was added to the culture in comparison with culture grown without pesticide.

DFZ =difenoconazole, EPZ = epoxyconazole, PNZ =penconazole, TBZ = tebuconazole, MTZ = metconazole, BMZ = bromuconazole, PTZ= prothioconazole, FCZ=fluconazole
